# Analysis of CCR2 splice variant expression patterns and functional properties

**DOI:** 10.1186/s13578-022-00787-6

**Published:** 2022-05-12

**Authors:** Hee-Kyung Park, Yun Hee Na, Huong Thi Nguyen, Lan Phuong Nguyen, Sunghoon Hurh, Jae Young Seong, Cheol Soon Lee, Byung-Joo Ham, Jong-Ik Hwang

**Affiliations:** 1 Department of Biomedical Sciences, College of Medicine, Korea University, 73 Goryeodae-ro, Seongbuk-gu, Seoul, 02841 Republic of Korea; 2 Department of Psychiatry, College of Medicine, Korea University, 73 Goryeodae-ro, Seongbuk-gu, Seoul, 02841 Republic of Korea

**Keywords:** CCR2, Splice variant, MCP-1, β-Arrestin, Chemotaxis

## Abstract

**Background:**

C–C motif chemokine receptor 2 (CCR2), the main receptor for monocyte chemoattractant protein-1 (MCP-1), is expressed on immune cells, including monocytes, macrophages, and activated T cells, and mediates cell migration toward MCP-1 in inflammation-related diseases. The *CCR2* gene encodes two isoforms: CCR2A and CCR2B. The CCR2B open reading frame is localized in a single exon, similar to other chemokine receptors, and CCR2A and CCR2B feature different amino acid sequences in their C-terminal intracellular loops due to alternative splicing. Most biochemical studies on CCR2-related cellular responses in the immune system have focused on CCR2B, with few reports focused on CCR2A. Understanding the functional properties of CCR2A in cellular responses may elucidate the roles played by MCP-1 and CCR2 in pathophysiological responses.

**Results:**

CCR2 gene expression analysis in several cell types revealed that most adherent cells only expressed CCR2A, whereas CCR2B expression was dominant in monocytic cells. The C-terminal Helix 8 region of CCR2A contains few basic amino acids, which may be unfavorable for cell surface localization, as confirmed with the HiBiT assay. CCR2B contains many C-terminal Ser/Thr residues, similar to other chemokine receptors, which may be phosphorylated by G protein–coupled receptor kinases (GRKs) to promote β-arrestin recruitment and subsequent endocytosis. By contrast, CCR2A contains few C-terminal Ser/Thr residues, which are unlikely to be phosphorylated by GRKs. CCR2A localized on the cell surface is resistant to internalization, despite the interaction between Gβ and GRKs induced by ligand binding with CCR2A. CCR2A induced cellular responses at a relatively higher degree than CCR2B, although both receptors mediated signaling events through Gαq and Gαi. HeLa cells lacking CCR2A showed slowed growth compared with parent cells, regardless of MCP-1 stimulation, and their chemotactic activity toward MCP-1, in addition to basal motility, was significantly impaired.

**Conclusion:**

MCP-1 and CCR2 may play pivotal roles in cancer progression by recruiting macrophages into cancer tissue. This study demonstrates that CCR2A but not CCR2B is expressed in solid cancer–derived cells. CCR2A is resistant to internalization by β-arrestin due to a distinct C-terminal region from CCR2B, which enhances MCP-1-stimulated responses, indicating that CCR2A may play essential roles in solid cancer progression.

**Supplementary Information:**

The online version contains supplementary material available at 10.1186/s13578-022-00787-6.

## Background

Chemokines refer to a family of small cytokines, approximately 8–10 kDa in size, that induce the migration of cells expressing chemokine receptors [1]. Chemokines are involved in physiological processes, such as tissue maintenance and development, and in immune responses, including immune surveillance and inflammation [2]. Chemokine receptor activation can induce various cellular responses, including proliferation, differentiation, angiogenesis, invasion, and metastasis [3, 4]. To date, more than 40 human chemokines have been identified, categorized into four subfamilies: two main groups (C–C motif chemokine ligand [CCL] and C–X–C motif chemokine ligand [CXCL]) and two minor groups (C–X3–C motif ligand [CX3CL] and C motif ligand [XCL]), based on the positions of conserved cysteine residues in the N-terminal region that form intramolecular disulfide bonds with other conserved cysteine residues [5]. Chemokine receptors consist of 18 members, classified into 10 CCL receptors (CCRs), 6 CXCL receptors (CXCRs), 1 CX3CL receptor (CX3CR), and 1 XCL receptor (XCR), according to their chemokine-binding properties [6]. Recently, G protein–coupled receptor 15 (GPR15) was deorphanized through the characterization of binding with a novel chemokine-like peptide [7]. In addition, four atypical chemokine receptors have been identified that bind chemokines but display different signaling properties from authentic receptors [8]. In many cases, several different chemokines bind to a single receptor to stimulate or inhibit receptor activity. In addition, a single chemokine can bind to several different receptors. These promiscuous binding properties between chemokines and their receptors may confer diverse or integrative biological responses, depending on their cellular or tissue expression patterns [9].

The open reading frames (ORFs) of most chemokine receptor genes are localized within a single exon, similar to other rhodopsin family G protein-coupled receptors (GPCRs). However, the alternative splicing of precursor mRNA can result in multiple receptor isoforms. Alternative splicing allows an organism to manage vital phenomena with a limited number of genes, and the alternative splicing of chemokine genes is likely to be involved in the regulation of cellular responses. For example, the *CXCR3* gene produces three splice variants: CXCR3B (415 aa) is encoded by a single exon, whereas CXCR3A (368 aa) and CXCR3Alt (267 aa) are derived from two exons through the alternative splicing of the N-and C-terminal regions, respectively. These variants activate different signaling pathways through biased agonism and tissue- or cell type-specific expression patterns [10]. CXCR3Alt, which contains five transmembrane domains and a short C-terminal region, likely acts as a decoy receptor [10]. Differences in the N-terminal lengths between CXCR3A and CXCR3B likely confer different properties and cellular responses. During cancer progression, CXCR3A is thought to play an important role in tumor metastasis, with relatively high expression. By contrast, CXCR3B is typically detected at much lower levels than CXCR3A, and CXCR3B overexpression appears to inhibit cancer progression [11].

The *CCR2* gene expresses two splice variants, CCR2A (374 aa) and CCR2B (360 aa) [12]. The CCR2B ORF is localized in a single exon, whereas the C-terminal loop of CCR2A is derived from a second exon through alternative splicing, resulting in different C-terminal loop regions between the two variants. CCR2 is the main receptor for monocyte chemoattractant protein-1 (MCP-1) and is expressed on various immune cells, including monocytes, macrophages, and activated T cells [13]. CCR2 plays pivotal roles in the immune system, mediating monocyte and macrophage recruitment to inflammatory sites and monocyte release from bone marrow [13]. CCR2 is among the most extensively studied chemokine receptors, with reports describing roles in various inflammatory diseases, ranging from infection and atherosclerosis to cancer. However, few studies examined the different roles played by the two splice variants. These proteins have been upregulated in different cell types in idiopathic inflammatory myopathies [14], suggesting potentially different roles of these variants.

In the present study, we demonstrated the effects of the different C-terminal tails associated with the two CCR2 isoforms on their molecular behaviors and functions. Although mRNA encoding both isoforms was detected in immune cell lines, nonimmune adherent cells expressed only CCR2A, like a previous report [15]. Functional assays comparing the ligand-stimulated cellular responses mediated by CCR2A and CCR2B revealed that unique functional behaviors of the variants are associated with the different C-terminal intracellular regions. Unlike immune cells, which predominantly express CCR2B, many cancer cells only express CCR2A, which may play a pivotal role to maintain their malignancy.

## Results

### CCR2 splice variant expression patterns

**Fig. 1:**
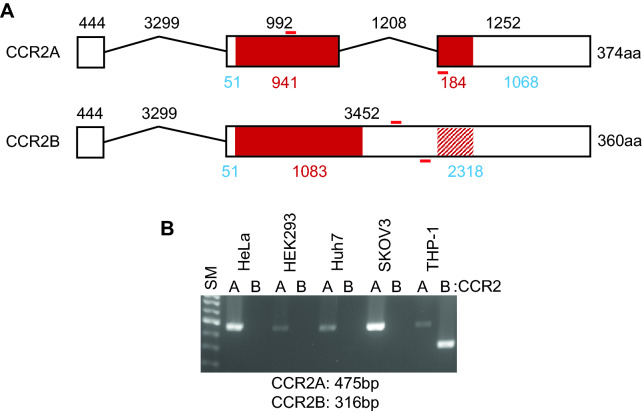
Schematics showing the *CCR2* gene structure and mRNA expression. **A** Localization of exons (dark red) and introns in the CCR2 splice variants. Red lines designate the primer sites used for each variant. **B** Reverse transcriptase-polymerase chain reaction (RT-PCR). Total RNA from cell lines was subjected to RT-PCR with splice variant-specific primers (shown below). PCR products were verified on 1.5% agarose gel. SM: 1 kb-plus ladder

The ORFs for most chemokine receptor genes are located within a single exon; however, some chemokine receptor genes feature alternative splicing that combines the ORF across two exons, resulting in the expression of multiple isoforms. *CCR2* has been cloned as two isoforms: *CCR2A* and *CCR2B*. The ORF for *CCR2B* is located within a single exon, similar to the gene structure for most chemokine receptors, whereas the ORF for *CCR2A* is separated across two exons, joined through alternative splicing, resulting in two *CCR2* isoforms that feature different C-terminal intracellular regions (Fig. 1A). To explore the expression pattern of these two *CCR2* isoforms, reverse transcriptase–polymerase chain reaction (RT-PCR) was performed using isoform-specific primer pairs in several cell lines. THP-1 cells, a human acute monocytic leukemia cell line, expressed both isoforms, with CCR2B mRNA detected at higher levels than CCR2A mRNA, which is similar to previous reports [14, 16]. All anchored cell lines tested here only expressed CCR2A mRNA (Fig. 1B), suggesting that the splicing machinery for this gene is highly active in these cells. Both isoforms resulted in identical amino acid sequences from the N-terminus to the seventh transmembrane domain, with the only differences observed in the C-terminal intracellular tail region.

### Interaction with β-arrestins internalized CCR2B but not CCR2A

**Fig. 2:**
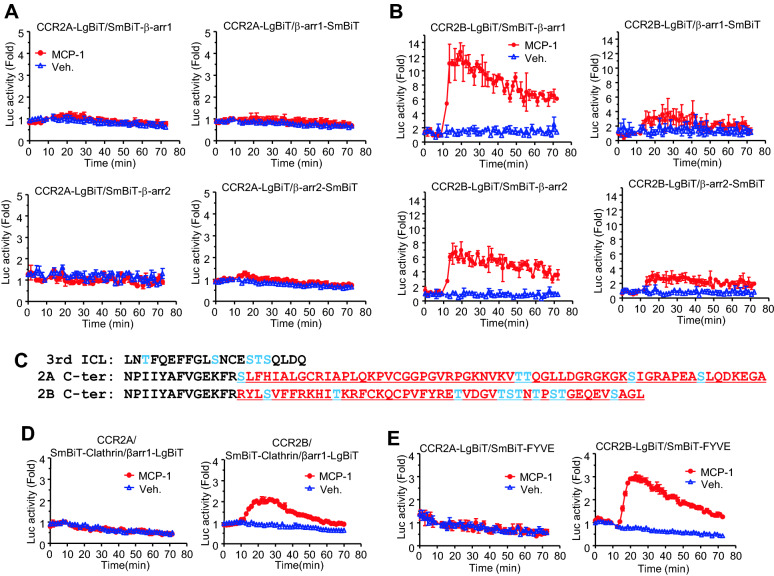
Analysis of receptor interactions with β-arrestins or endosomal markers using the NanoBiT assay. C-terminal LgBiT forms of CCR2A (**A**) or CCR2B (**B**) were co-expressed with β-arrestin 1-SmBiT or β-arrestin 2-SmBiT in HEK293 cells. The cells were incubated with the luciferase substrate and treated with MCP-1. Luciferase activity was measured with a luminometer for 70 min. The graphs are representatives of more than three independent experiments. **C** The amino acid sequences of the third intracellular loop and C-terminal regions of the variants. Blue colors indicate Ser/Thr residues. Red colors indicate the amino acid sequences that differ between the variants. **D** HEK293 cells expressing SmBiT-Clathrin and b-arrestin1-LgBiT with each of CCR2 variants were treated with 100 ng/ml MCP-1 and the luminescence change was measured. **E** HEK293 cells co-expressing the C-terminal LgBiT forms of the CCR2 variants and SmBiT-FYVE were treated with 100 ng/ml MCP-1 and the luminescence change was measured

In addition to G protein activation, most chemokine receptors interact with β-arrestins, which induce internalization to downregulate their chemokine signaling and promote β-arrestin-mediated signaling. Using a structural complementation analysis based on NanoBiT technology, the interactions between β-arrestins and CCR2A or CCR2B were investigated. Multiple combinations of NanoBiT constructs featuring the receptor isoforms and β-arrestins were expressed in HEK293 cells treated with MCP-1, and luciferase activity was measured using Nano-Glo Live Cell Reagent and a luminometer. Cells expressing any NanoBiT construct combination of CCR2A and β-arrestin showed no increase in luciferase activity following MCP-1 treatment (Fig. 2A), whereas MCP-1 treatment induced significant luciferase activation for the combination of CCR2B-LgBiT and SmBiT-β-arrestin 1/2 (Fig. 2B). Maximum luciferase activity was observed for the interaction between CCR2B and β-arrestin 1, which was approximately twice as high as that observed for β-arrestin 2, implying that CCR2B has a higher affinity for β-arrestin 1.

Many GPCRs require GPCR kinase (GRK)-dependent Ser/Thr phosphorylation of the third intracellular loop and the C-terminal region to interact with β-arrestin. Of the ten Ser/Thr residues identified in the C-terminal region of CCR2B, some, especially in Ser/Thr-rich sequence, may potentially act as target sites for GRK phosphorylation, whereas none of the five Ser/Thr residues in the C-terminal region of CCR2A are likely to be phosphorylated by GRKs (Fig. 2C). Although a few common Ser/Thr residues were identified in the third intracellular loop between these two variants, these may be neither phosphorylated by GRKs nor sufficient for the phosphorylation-dependent recruitment of β-arrestin.

CCR2A is 14 amino acids longer in the C-terminal region than CCR2B, which might contribute to differences in their biological roles (Fig. 2C). The interaction with β-arrestins facilitates the internalization of GPCRs [17]. To examine the internalization behavior of the CCR2 variants, SmBiT-Clathrin and b-arrestin1-LgBiT were co-expressed with CCR2 variants in HEK293 cells, and MCP-1-stimulated luciferase activity was measured (Fig. 2D). Furthermore, NanoBiT constructs of the receptors and the FYVE domain from early endosome antigen 1 (EEA1), an early endosomal marker, were expressed in HEK293 cells, and MCP-1-stimulated luciferase activity was measured (Fig. 2E). Both data showed that MCP-1-dependent luciferase activity was increased in CCR2B-expressing cells but not in CCR2A-expressing cells, suggesting that only CCR2B is internalized to endosome in a β-arrestin-dependent manner.

### Both CCR2 variants mediate the MCP-1-stimulated interaction between Gβ1 and GRKs

**Fig. 3:**
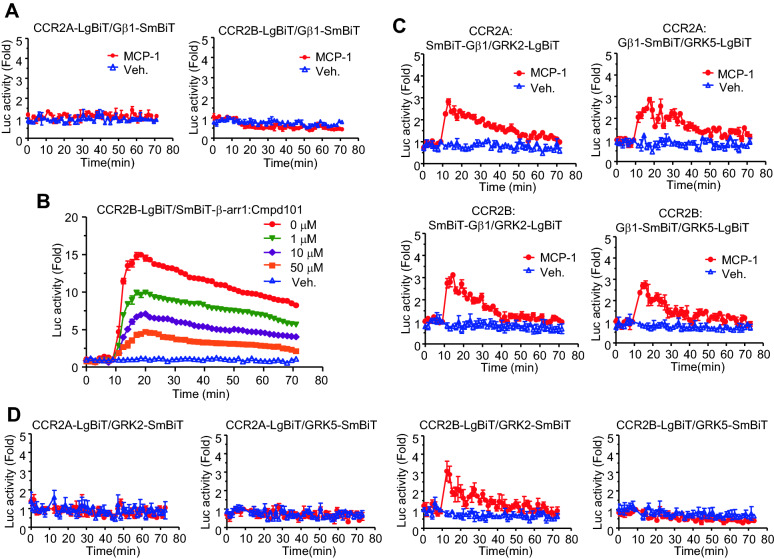
MCP-1–dependent interactions between Gβ1 and G protein–coupled receptor kinases (GRKs) were mediated by both splice variants. **A** HEK293 cells expressing CCR2-LgBiT and Gβ1-SmBiT were treated with monocyte chemoattractant protein-1 (MCP-1). Molecular interaction was assessed by measuring luciferase activity. **B** Cells expressing CCR2B-LgBiT and SmBiT-β-arrestin 1 were pretreated with different doses of Cmpd101, a GRK2/3 inhibitor, and then stimulated with MCP-1 in the presence of luciferase substrate. **C** Cells expressing CCR2B or CCR2A with different Gβ1 and GRK NanoBiT constructs were treated with MCP-1, and luciferase activity was detected with a luminometer. **D** MCP-1-dependent interaction between CCR2B and GRK2. Cells co-expressing the NanoBiT forms of CCR2A or CCR2B together with GRK2 or GRK5 were treated with MCP-1, and luciferase activity was measured

Following ligand binding, CCR2 interacts with and activates heterotrimeric G proteins, resulting in the dissociation of Gα and Gβγ subunits, which activate distinct signaling pathways. However, the interaction between the CCR2 isoforms and either Gα or Gβγ could not be confirmed using the NanoBiT assay, despite the examination of all possible combinations (Fig. 3A, and data not shown). β-arrestins are recruited to CCR2B through interactions with GRK-phosphorylated regions. Seven GRK isoforms have been identified and can be divided into three subfamilies based on their functions, structures, and expression patterns [18]. GRK2/3 and GRK5/6 are ubiquitously expressed in mammalian tissues and, therefore, are considered likely to regulate the activity of most GPCRs [19]. To confirm involvement of GRK2/3 in β-arrestin recruitment to CCR2B as previously described [17, 20], cells expressing NanoBiT constructs for CCR2B and β-arrestin 1 were pretreated with a GRK2/3-specific kinase inhibitor, Cmpd101, and MCP-1-dependent luciferase activity was measured. As shown in Fig. 3B, the luciferase activity stimulated by MCP-1 declined in a Cmpd101 dose–dependent manner. However, treatment with 50 µM Cmpd101, which was sufficient to complete inhibition of GRK2/3 activity [21], did not inhibit all luciferase activity, suggesting that other GRK subfamilies may contribute to CCR2B phosphorylation.

Gβγ separated from the GTP form of Gα may binds to GRKs to make it easier to recognize and phosphorylate ligand bound GPCRs [22]. To examine the interaction between Gβ1 and GRKs, a real-time luciferase assay was performed in HEK293 cells expressing NanoBiT constructs for both proteins. In the presence of CCR2A and CCR2B, MCP-1 stimulated luciferase activities through binding of SmBiT-Gβ1 with either of GRK2-LgBiT or GRK5-LgBiT (Fig. 3C), suggesting that the interaction between GRK2 and Gβ1 differs from that of GRK5 and Gβ1. These findings suggest that MCP-1 binding to both CCR2A and CCR2B triggers the dissociation of Gα and Gβγ, resulting in the subsequent interaction between Gβ and GRKs.

To investigate whether the chemokine receptors interact with GRKs, cells expressing different combinations of NanoBiT constructs were treated with MCP-1 and subjected to a real-time luciferase assay. The ligand-dependent luminescence increase was observed in cells expressing CCR2B-LgBiT and GRK2-SmBiT, suggesting that GRK2 may interact with CCR2B through Gβγ. However, no signal change was observed for any combination of CCR2B and GRK5, suggesting that this assay system may not be suitable for examining the interaction between CCR2B and GRK5. No ligand-stimulated luciferase activity was observed for any combination between CCR2A and either GRK2 or GRK5 (Fig. 3D, Additional file 1: Fig. S1), providing additional evidence that the C-terminal region of CCR2A may not interact with GRKs or support GRK-mediated phosphorylation.

### The C-terminal tail region may play an essential role in the membrane localization of CCR2

**Fig. 4:**
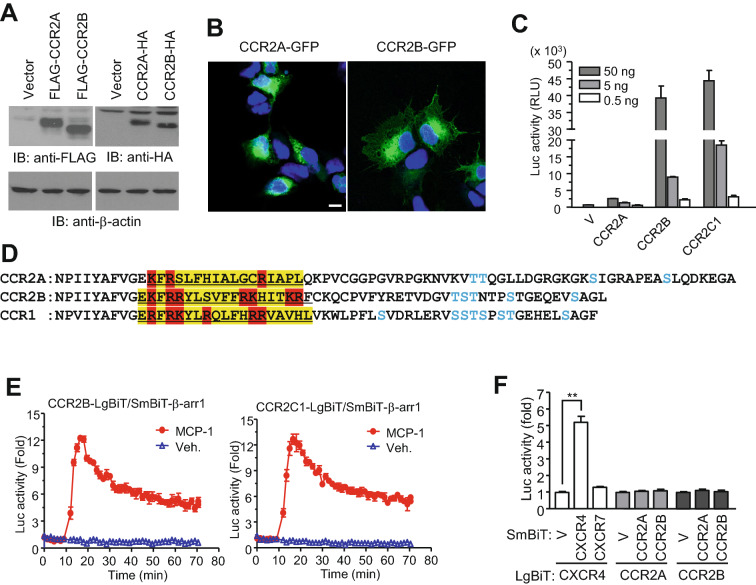
Membrane localization of CCR2 variants. **A** HEK293 cells were transfected with plasmids containing FLAG or HA-tagged *CCR2* variant sequences and lysed with radioimmunoprecipitation assay (RIPA) buffer. The clarified cell extracts were subjected to western blotting using the appropriate antibodies. **B** HEK293 cells on coverslips were transfected with GFP-tagged forms of CCR2 variants and fixed with 4% paraformaldehyde, and the GFP signals were observed under a confocal microscope. **C** Different quantities of plasmids containing the N-terminal SmBiT-tagged chemokine receptor sequences were transfected in HEK293 cells. HiBiT activities were measured by a luminometer at a single time point. CCR2C1: the chimeric form of CCR2 in which the C-terminal region was replaced with the C-terminal region from CCR1. **D** Helix 8 in the C-terminal regions of CCR2A, CCR2B, and CCR1. Yellow color designates Helix 8. The red color indicates basic amino acids. **E** Cells co-expressing SmBiT-β-arrestin 1 with CCR2B-LgBiT or CCR2C1-LgBiT were treated with monocyte chemoattractant protein-1 (MCP-1), and luciferase activity was measured by a luminometer. **F** The basal luciferase activity was measured in cells expressing both the SmBiT and LgBiT forms of the receptors

The detection of endogenous CCR2 proteins using biochemical methods can be difficult because of protein amount, difficulty of 7-TM protein preparation, and lack of appropriate antibodies for detection; therefore, plasmids expressing epitope-tagged CCR2 genes were generated. CCR2A and CCR2B were expressed in HEK293 cells with N-terminal FLAG-tags or C-terminal HA-tags, and cell lysates were subjected to western blot analysis using appropriate antibodies. As shown in Fig. 4A, regardless of the epitope position, both isoforms were expressed at similar levels. However, confocal images of C-terminal GFP-tagged receptors demonstrated that CCR2A was primarily localized to the cytosol, whereas CCR2B signals were detected in the plasma membrane (Fig. 4B), suggesting that the C-terminal region determines the membrane localization of chemokine receptors. The membrane localization of chemokine receptors was further determined by the HiBiT assay. Luminescence signals in cells expressing N-terminal SmBiT-tagged receptors became stronger depending on the transfected plasmid amount. Intriguingly, SmBiT-CCR2B signals were much stronger than those for SmBiT-CCR2A, which was consistent with the plasma membrane localization patterns observed using GFP-tagged receptors, as shown in Fig. 4B. Comparing the sequences of the C-terminal region revealed that fewer basic amino acids were located in the membrane-proximal region of CCR2A than in a similar region of CCR2B (Fig. 4C). Other chemokine receptors (e.g., CCR1) also contain a relatively higher number of basic amino acids in similar regions. To further explore the specific roles played by basic amino acids in determining the membrane localization of the receptor, an SmBiT-fusion CCR2 chimera was generated, in which the C-terminal region was replaced with the C-terminal region from CCR1 (CCR2C1). In the HiBiT assay, luciferase activity induced by the chimera was similar to that induced by SmBiT-CCR2B (Fig. 4D). Furthermore, both CCR2B and CCR2C1 recruited β-arrestin 1 following MCP-1 stimulation with similar efficiencies (Fig. 4E). These results suggest that the C-terminal region may play a pivotal role in determining the plasma membrane localization of chemokine receptors and β-arrestin recruitment and that the C-terminal CCR2A region may be insufficient for membrane localization. Many GPCRs are expressed at the cell surface as homodimers or heterodimers. In previous studies using NanoBiT technology, homodimerization but not heterodimerization was observed for CXCR4 and CXCR7. However, the NanoBiT assay performed using cells expressing CCR2A and CCR2B, as SmBiT or LgBiT constructs, showed no increases in luminescence signals, implying that CCR2 isoforms are not able to form homodimeric receptor complexes (Fig. 4F).

### Comparison of CCR2 splice variant-mediated G protein signaling

**Fig. 5:**
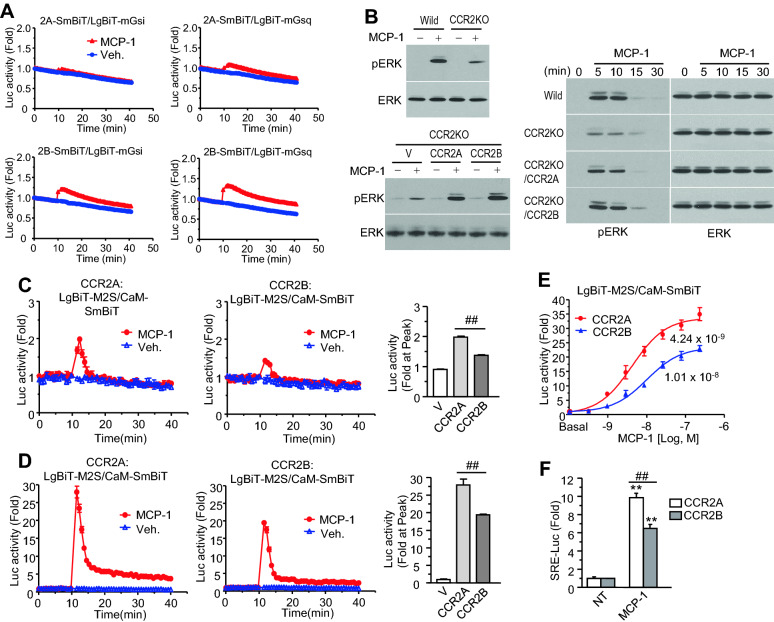
Analysis of the cellular responses mediated by CCR2A or CCR2B. **A** Specific interaction between CCR2 variants and Gα isotypes. HEK293 cells expressing C-terminal SmBiT-tagged receptors and N-terminal LgBiT-tagged mini-G proteins were treated with MCP-1 and luciferase activity was measured by a luminometer. **B** ERK phosphorylation. Wild type cells and CCR2 KO cells were treated with MCP-1 for 10 min (left upper panel). CCR2 KO cells and CCR2 variant-reconstituted cells were treated with MCP-1 for 10 min (left lower panel). All cells described in the figure were treated with MCP-1 for different time (right panel). Cell extracts were subjected to western blotting with anti-phospho-extracellular signal-regulated kinase (pERK) antibodies or anti-ERK antibodies. **C**, **D** Intracellular Ca^2+^ measurements assessed using the enzyme complementation assay. **p > 0.01 (**C**, **D**) HEK293 cells (**C**) or HEK293-Gqi cells (**D**) expressing LgBiT-MYLK2s (described as M2S) and calmodulin-SmBiT (described as CaM) together with CCR2A or CCR2B were treated with MCP-1, and luciferase activity was determined using a luminometer. The graph on the right side shows the maximum fold change in luciferase activity induced by MCP-1. ##p > 0.01, in comparison between CCR2A and CCR2B. V: vector (**E**) MCP-1 dose dependency of the calcium responses. HEK293-Gqi cells expressing the calcium probes with receptors were treated with different doses of MCP-1. The graph shows the maximum fold change in luciferase activity. The numbers below each curve designate EC_50_ value (M) of MCP-1. **F** Reporter gene assay. Cells containing the Gqi construct were transfected with the serum response element-luciferase (SRE-Luc) gene and CCR2 variants. After overnight starvation, the cells were treated with MCP-1 for 6 h and lysed in lysis buffer. The luciferase activity of each extract was measured using a luminometer. NT: non-treated. ##p > 0.01, difference between CCR2A and CCR2B. **p > 0.01, compared with non-treated. All experiments were conducted at least three independent times

To explore the cellular responses mediated by CCR2 splice variants, downstream signaling events were evaluated. Recently, engineered G proteins, called mini-G proteins (mG), were developed to study the biophysical interactions between active GPCRs and each subtype of G proteins [23, 24]. We constructed mG proteins in NanoBiT vectors, as described in previous reports. The analysis of different combinations of LgBiT- and SmBiT-tagged proteins revealed that N-terminal-tagged LgBiT constructs for each mG protein are likely to bind with CCR2-SmBiT constructs (data not shown). In general, chemokine receptors activate Gαi/o, Gα16, or Gα12/13. MCP-1 induced luminescence in cells expressing mGsi and each of the CCR2 variants, particularly CCR2B (Fig. 5A). Unfortunately, luminescence signals in the presence of either mG16 or mG12 did not increase with MCP-1 stimulation (data not shown), indicating that these mini-G proteins may not provide an adequate structure to interact with activated receptors, as shown in previous reports [23, 24]. The luminescence signals were increased by interaction between mGsq and both CCR2 variants in the presence of MCP-1, which confirms CCR2-mediated activation of both Gαi and Gαq pathways [25, 26].

Extracellular signal-regulated kinase (ERK) phosphorylation is a representative event in GPCR-mediated signaling pathways [27]. MCP-1-stimulated ERK phosphorylation was detected in HEK293 cells without the expression of exogenous CCR2 variants, and according to RT-PCR analysis, CCR2A is endogenously expressed in HEK293 cells (Fig. 1B). To determine the effects of each CCR2 variant on MCP-1-stimulated ERK phosphorylation, CCR2 knockout (KO) cells were developed using the CRISPR-Cas9 system with subsequent cloning and genomic DNA analysis. ERK was weakly phosphorylated following MCP-1 stimulation in CCR2 KO cells compared to parental cells (Fig. 5B). Although CCR2 is the only GPCR known to bind MCP-1, MCP-1 has been reported to interact with glycosaminoglycans (GAGs), a group of cell surface proteins [23, 24] which may be responsible for the MCP-1-stimulated ERK phosphorylation observed in the absence of CCR2. MCP-1-stimulated ERK phosphorylation was enhanced by the expression of exogenous CCR2A and CCR2B in CCR2 KO cells, suggesting that both variants have equal impacts on ERK phosphorylation. The observed time-dependent phosphorylation pattern was similar in cells expressing wild-type CCR2, CCR2 KO cells, and cells expressing exogenous CCR2A or CCR2B, appearing at 5 min, declining at 15 min, and disappearing at 30 min.

MCP-1 appears to activate Gαq as well as Gαi through CCR2 variants (Fig. 5A), and an increase in intracellular calcium was determined by NanoBiT technology using LgBiT-MYLK2s (calmodulin-binding motif in myosin light-chain kinase 2) and calmodulin-SmBiT, which were previously developed to measure real-time intracellular calcium change in our laboratory [28]. MCP-1-stimulated luminescence increased slightly in cells expressing CCR2A and CCR2B, with a stronger signal in cells expressing CCR2A than in cells expressing CCR2B (Fig. 5C). Since chemokine receptors mainly activate the Gαi/o pathway often activate calcium signaling, cells expressing the chimeric G protein Gαqi which converts Gαi to Gαq pathway were subjected to the NanoBiT calcium assay to determine activation of the pathway. The luminescence induced by MCP-1 treatment was much higher than that observed in the absence of Gαqi (Fig. 5D), and the CCR2A-mediated signals were stronger than those mediated by CCR2B, which increased in an MCP-1 dose-dependent manner (half-maximal excitatory concentration: 4.24 × 10^−9^ for CCR2A vs. 1.01 × 10^−8^ for CCR2B; Fig. 5E, Additional file 1: Fig. S2). Downstream signals of ERK phosphorylation and calcium upregulation can stimulate transcription factor binding to the serum response element (SRE); therefore, SRE-reporter gene assay was performed in cells expressing CCR2A and CCR2B. Figure 5F shows that CCR2A mediated stronger luciferase activity than CCR2B, which was consistent with the results of the calcium assay. These results indicate that CCR2A mediates MCP-1-stimulated G protein signaling with relatively high potency compared with CCR2B.

### CCR2A is essential for cancer cell proliferation and migration

**Fig. 6:**
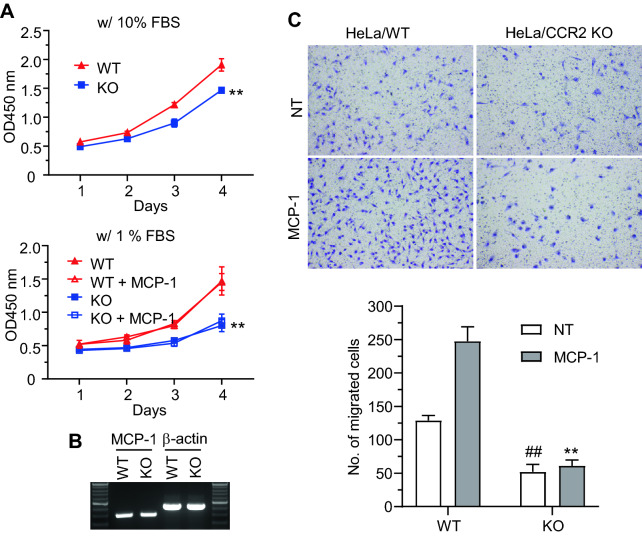
CCR2A is involved in the proliferation and migration of HeLa cells. **A** Wild-type (WT) and CCR2A knockout (KO) HeLa cells were seeded in 96-well plates with medium supplemented with 10% fetal bovine serum (FBS, upper graph) or medium supplemented with 1% FBS and 100 ng/ml monocyte chemoattractant protein-1 (MCP-1, lower graph). Cells were subjected to cell counting kit 8 (CCK-8) assay every 24 h for 4 days. **p > 0.01 compared with WT cells. **B** Reverse transcriptase–polymerase chain reaction (RT-PCR) for MCP-1 gene with total RNAs isolated from WT or CCR2A KO HeLa cells. β-actin was used as a control. **C** Transwell migration assay. Cells were loaded in the upper chamber at a density of 1 × 10^4^ cells/well, and 100 ng/ml MCP-1 was added to the lower chamber. After 24 h, the transmembrane was fixed, the remaining cells in the upper wells were removed, and the migrated cells were stained. The cells that migrated to the lower surface of the transmembrane were counted in four different areas. ##p > 0.01 compared with WT, non-treated (NT), **p > 0.01 compared with WT treated with MCP-1. All experiments were conducted at least three independent times

To explore the functional effects of CCR2 signaling on cellular behavior, CCR2 KO HeLa cells were generated using the CRISPR-Cas9 system, and their growth was compared with that of parent cells. Under normal culture conditions containing 10% fetal bovine serum (FBS), the growth rate of the KO cells was slightly slower than that of the parent cells (Fig. 6A, upper graph). To examine the effects of MCP-1 treatment on cell growth, the cells were treated with 100 ng/ml MCP-1 in the presence of 1% FBS because HeLa cells die easily in the absence of serum. CCR2 KO cells grew slowly compared with their parent cells, regardless of MCP-1 treatment (Fig. 6A lower graph). RT-PCR using specific primer pairs revealed similar levels of endogenous MCP-1 expression in both cell groups (Fig. 6B), suggesting that the lack of response to exogenous MCP-1 application might be due to the strong endogenous expression of MCP-1 in HeLa cells. In a migration assay, CCR2 KO HeLa cells did not migrate to the lower chamber containing MCP-1, suggesting that the chemotactic activity of HeLa cells toward MCP-1 was impaired in the absence of CCR2. The spontaneous motility of CCR2 KO cells was also decreased significantly in media containing 1% FBS (Fig. 6C).

## Discussion

Several reports have demonstrated the roles played by CCR2 in immune cell functions, including monocytes, macrophages, and T cells, during inflammatory conditions [13]. The absence of CCR2 reduces inflammation and alters the differentiation of some T cell lineages [29–31]. Although the existence of splice variants has been acknowledged, most studies have focused on the overall role of CCR2 in the immune system without exploring the unique functions of the different variants. The structures of both variants were analyzed by X-ray crystallography due to the physiological significance of CCR2, and differences between the two variants were only resolved in Helix 8 next to transmembrane domain 7 in the C-terminal intracellular region; however, this analysis was unable to delineate the unique properties of these differential regions [32, 33]. To characterize the functional differences between these variants, their mRNA expression levels were assessed in several cell lines. According to RT-PCR, monocytic cells may express both isoforms, with CCR2B being the dominant form, suggesting that the MCP-1 response in monocytes may be mediated by CCR2B. Interestingly, all of the anchored cells we tested expressed only *CCR2A* mRNA, indicating that CCR2A may function as an MCP-1 receptor in the majority of non-immune cells and tissues. The cells we examined are all cancer cell lines, and their gene expression patterns may be biased. There is a report that described mRNA expression of both CCR2A and CCR2B in inflammatory muscle tissues, implying that CCR2B may be expressed in solid tissue. However, it is hard to exclude the possibility that the transcript was from immune cells because they examined with inflammatory tissues [14]. Our further RT-PCR revealed that only CCR2A mRNA was expressed in human aortic endothelial cells (data not shown). Therefore, gene expression should be further examined in normal tissues or cells to confirm the previous expression pattern. However, because *CCR2A* mRNA can be detected in HEK293 cells, which are immortalized cells but are not cancer cells, these proteins are likely expressed in various cells or tissues at relatively low levels.

According to the Ensembl genome analysis database (http://www.ensembl.org/), the splicing process used to produce CCR2A may be unique in some primate species. Both transcripts were identified in humans, but only the CCR2A transcript was identified in chimpanzees. By contrast, only CCR2B appears in other species, including monkeys. This finding suggests that CCR2A may be necessary for specific evolutionary biological roles separate from those played by CCR2B in humans and chimpanzees. Unfortunately, mice cannot be used to explore the tissue expression and biological functions of CCR2A because the mouse *CCR2* gene does not undergo the alternative splicing process necessary to generate CCR2A.

The alternative splicing of the human *CCR2* gene produces two proteins with different C-terminal intracellular regions. The NanoBiT assay revealed that only CCR2B was internalized by interactions with β-arrestin 1/2 in the presence of MCP-1. β-arrestin 1/2 is thought to bind with phosphorylated Ser/Thr residues in the third intracellular loop and the C-terminal regions of GPCRs [34]; therefore, even though the consensus sequences targeted by GRKs are not clear, the enriched and consecutive Ser/Thr residues in CCR2B are likely phosphorylated by GRKs and targeted by β-arrestins [17, 20], whereas the few Ser/Thr residues in CCR2A may not be phosphorylated by GRKs. Ligand binding of chemokine receptors results in the dissociation of heterotrimeric G proteins, and the released Gβγ subunit binds to GRKs and enables the phosphorylation of activated receptors [35]. MCP-1 stimulated the interaction between Gβ1 and GRKs in the presence of either receptor, implying that even CCR2A activates heterotrimeric G proteins and releases Gβγ. However, CCR2B but not CCR2A interacted with GRK2 in the enzyme complementation assay, suggesting that CCR2B is a substrate of GRK2. GRK5 could also phosphorylate CCR2B, but their interaction was not detected in the enzyme complementation assay. Although the enzyme complementation assay is a powerful read-out for the detection of protein–protein interactions, not all interactions are able to be verified using this technology because molecular interactions and steric conformations may affect the activity induced by the interaction between two enzyme fragments. Although this technical limitation can sometimes be overcome through the use of different N-terminally or C-terminally tagged forms, not all proteins are suitable for this assay. The results revealed that the C-terminal CCR2A region is unfavorable for GRK phosphorylation, which is necessary for β-arrestin interaction and subsequent receptor internalization.

Western blot analyses of total cell lysates showed that the protein expression of the variants was not affected by their C-terminal intracellular regions. However, the cellular fluorescence observed using GFP-tagged receptors and results from the HiBiT assay indicated that the cell surface localization of these receptors is likely affected by the amino acid sequence of the C-terminal region. Helix 8 may be particularly important for the proper membrane localization of chemokine receptors [36]. According to a secondary structure prediction program for proteins (http://www.compbio.dundee.ac.uk/jpred4/index_up.html), both CCR2A and CCR2B contain Helix 8, which consists of approximately 18 amino acids proximal to the seventh transmembrane domain. However, more basic amino acids are found in the helix structure of CCR2B compared with CCR2A, which may influence the stable membrane attachment of the helix by altering the interaction with phospholipids. Similar to CCR2B, efficient plasma membrane localization and an equivalent level of MCP-1-mediated β-arrestin 1 recruitment was observed for CCR2C1, a chimeric receptor in which the C-terminal region was replaced with the C-terminal region from CCR1, suggesting that the presence of basic and Ser/Thr residue in the C-terminal region have significant functional effects. Residues other than those found in Helix 8 may also be important for the membrane localization of GPCRs, as indicated by a report suggesting that the cytoplasmic retention signal may be located between residues 316 and 349 [12]. When CCR2A is localized to the cell surface, the unique amino acid sequence of the C-terminal region may enable the receptor to remain in the plasma membrane following MCP-1 stimulation, resulting in sustained signaling, which is supported by evidence from the reporter gene assay and intracellular Ca^2+^ responses. As shown in Fig. 5E and F, calcium influx and SRE-dependent luciferase expression stimulated by MCP-1 were significantly higher in the presence of CCR2A compared to CCR2B. Interestingly, in previous studies performed by Vatter et al., the ligand dependent SRE-driven luciferase expression was quite similar between cells expressing CCR2A and CCR2B [37]. The difference from our results may be due to expression system. COS-7 cells used in previous report are constitutively activated and can highly express exogenous proteins through an oncogene, Simian Virus 40 large T antigen, while HEK293 cells may express relatively low level of the receptor to distinguish the effect of CCR2A and CCR2B.

CCR2 and its cognate ligand MCP-1 have been extensively studied for their roles in the tumor microenvironment. MCP-1 is expressed in tumor cells, and the surrounding stromal cells recruit macrophages to tumor tissue, exacerbating the disease condition [38]. Recent studies have suggested that CCR2–MCP-1 signaling may promote cancer progression due to their overexpression in cancer cells [39]. According to the gene expression data, MCP-1-dependent cancer progression must be ascribed to CCR2A as a canonical pathway, even if the roles of some GAGs that bind MCP-1 in membrane and transduce signals could not be excluded in the process [20, 21]. The role played by CCR2A was also confirmed by the deletion of the receptor from HeLa cells, which resulted in impaired proliferation and migration activities compared with the parent cells.

## Conclusion

CCR2 splice variants showed a unique expression pattern in several cell lines, associated with differential functions and cellular responses compared with CCR2B, which is considered the dominant MCP-1 receptor involved in immune cell responses. However, CCR2A was primarily detected in non-immune cells, such as cancer cells that originated from epithelial and mesenchymal tissues, which may confer unique chemokine responses to these cells. Although the C-terminal region of CCR2A does not appear to promote the membrane localization of the receptor, membrane-localized CCR2A is still able to transduce chemokine stimulation to G proteins, and the lack of GRK-target residues makes CCR2A resistant to β-arrestin-mediated internalization, resulting in relatively stronger CCR2A-mediated signals compared with those mediated by CCR2B, suggesting the importance of CCR2A in cancer cell behaviors and progression.

## Materials and methods

### Materials

MCP-1 and other chemokines were purchased from Peprotech (Rocky Hill, NJ, USA). A NanoBiT starter kit containing the plasmids and all reagents for the protein interaction assay was purchased from Promega (Madison, WI, USA). pBiT3.1 plasmid and all reagents for these plasmid-related assays were also obtained from Promega. The SRE-Luc vector, which contains four copies of the SRE (CCATATTAGG), was acquired from Stratagene (La Jolla, CA, USA). All primers required for vector construction and related materials were obtained from Cosmo Genetech Co., Ltd. (Seoul, Korea), and DNA sequencing was conducted by Macrogen (Seoul, Korea). Anti-HA antibodies and anti-FLAG antibodies were obtained from Sigma-Aldrich (St. Louis, Mo, USA). Anti-ERK (cat. no. 4695) and anti-pERK (Thr202/Tyr204; cat. no. 4370) antibodies were obtained from Cell Signaling Technology (Beverly, MA, USA). Anti-β-actin (cat. no. sc-9996) and all secondary antibodies were obtained from Santa Cruz Biotechnology (Santa Cruz, CA, USA). Unless otherwise stated, all reagents were purchased from Sigma-Aldrich.

### Cell culture

HEK293, HeLa, and SKOV-3 cells obtained from the American Type Culture Collection (ATCC, Manassas, VA, USA) were maintained in Dulbecco’s modified Eagle medium (DMEM) supplemented with 10% FBS, 100 U/ml penicillin G, and 100 µg/ml streptomycin (Invitrogen, Carlsbad, CA, USA). Huh-7 and THP-1 cells obtained from ATCC were maintained in RPMI 1640 medium supplemented with 10% FBS, 100 U/ml penicillin G, and 100 µg/ml streptomycin. HEK293 Gαqi cells stably expressing chimeric Gαqi proteins, in which with the four C-terminal amino acids were replaced by those from Gαi2, were used for the intracellular Ca^2+^ assay.

### Establishment of CCR2 KO cell lines by CRISPR-Cas9

To establish cells lacking CCR2 expression, four potential target sequences were selected in the *CCR2* gene using a guide design program from the Zhang Lab (https://zlab.bio/guide-design-resources). Forward and reverse strand oligos for the target sequences were annealed and inserted into the pRG2 vector to express guide RNAs. A 49-nucleotide sequence including the target site and the surrounding sequences were inserted into the pMRS surrogate vector. The vectors were introduced into HEK293 cells with p3S-Cas9 plasmids, and the guide efficiency was assessed by genomic DNA PCR with appropriate primers and T7E1 treatment. Two efficient guide vectors (TGCTGTCCACATCTCGTTCT CGG and TTCACAGGGCTGTATCACAT CGG), a surrogate vector, and p3S-Cas9 were transfected into HEK293 or HeLa cells. Potential gene KO cells were isolated by MACSelect Kk MicroBeads (Miltenyi Biotec, Bergisch Gladbach, Germany) and transferred into 96-well plates at 0.5 cells/well. CCR2 KO was confirmed by genomic DNA PCR and T7E1 analysis.

### RT-PCR

All cells were cultured for 2 days after seeding in a 60-mm dish, washed with phosphate-buffered saline (PBS), and treated with TRIzol (Invitrogen). Total RNAs were isolated following the manufacturer’s instructions, and cDNAs were prepared using reverse transcriptase from Promega. Using the cDNAs, PCR proceeded through 30 cycles of primer annealing (59 °C for 30 s), extension (72 °C for 40 s), and denaturing (95 °C for 30 s) using the splice variant–specific primer pairs: CCR2A, F: TGGCTGTGTTTGCTTCTGTC and R: GCAATCCTACAGCCAAGAGC; CCR2B, F: AGTTTTGGTGGAGTCCGATG and R: GTCCTTTGCTCCTGGTGAAG. One-tenth of the PCR products were loaded onto a 1.5% agarose gel.

### *NanoLuc luciferase complementation (NanoBiT) assay for molecular interactions and intracellular Ca*^*2*+^*changes*

HEK293 cells were seeded on 96-well plates at a density of 2 × 10^4 ^cells/well. The next day, 50 ng of each plasmid, featuring N-terminal or C-terminal SmBiT or LgBiT tagged proteins, were mixed with 0.2 µl Lipofectamine 2000 (Invitrogen, Carlsbad, CA) in Opti-MEM and added to cells. The other transfection steps were performed according to the manufacture’s instruction. All two-gene combinations were transfected in a similar fashion. After 24 h, before measuring luminescence, the cells were stabilized for 10 min at room temperature by replacing the medium with 100 µl Opti-MEM. Then, 25 µl Nano-Glo Live Cell Reagent (furimazine) was added to each well, and baseline luminescence was measured for the first 10 min. The cells were then stimulated with 10 µl chemokine solution at a concentration of 100 ng/ml, and the cell plate was measured continuously for 1 h. These procedures were conducted using a luminometer (Synergy 2 Multi-Mode Microplate Reader: BioTek Instruments, Inc., Winooski, VT, USA). Receptor dimerization was investigated using the NanoBiT assay in cells expressing either the same receptors or different receptors, each tagged with SmBiT and LgBiT.

For the calcium assay, cells were plated in a 96-well plate and transfected with 30 ng of the receptor plasmid, 30 ng of the calmodulin-SmBiT plasmid, and 30 ng of the LgBiT-MYLK2s plasmid (calmodulin target sequence in MYLK2: LLKKYLMKRRWKKNFIAVSAANRFKK) using Lipofectamine 2000 and the remaining steps were as described above.

### HiBiT assay

The localization of receptors on the cell membrane was detected using the Nano-Glo HiBiT extracellular system (Promega, Madison, USA). HEK293 cells were seeded in 96-well plates at a density of 2.0 × 10^4^ cells per well. The next day, the cells were transfected with a mixture of DNA constructs containing SmBiT-tagged receptors and 0.2 µl Lipofectamine 2000. After 24 h, 100 µl Nano-Glo HiBiT extracellular reagent (1 µl LgBiT protein + 2 µl substrate + 97 µl of Nano-Glo HiBiT buffer) was added to each well. The assay plate was allowed to equilibrate to room temperature for 4 min without mixing, and then the luminescence was measured with a luminometer.

### Western blotting

HEK293 cells were transfected with plasmids containing C-terminal epitope-tagged CCR2A or CCR2B. After 36 h, the cells were harvested in radioimmunoprecipitation assay (RIPA) buffer (50 mM Tris–HCl pH 7.5, 150 mM NaCl, 1 mM EDTA, 1% sodium deoxycholate, 1% Triton X-100) containing protease inhibitor cocktail, and 20 µg of extracts were separated by sodium dodecyl sulfate–polyacrylamide gel electrophoresis, and western blotting was performed using antibodies against the epitopes. For the analysis of ERK phosphorylation, the cells were transfected with plasmids containing the splice variants, incubated with serum-free media overnight, and treated with 100 ng/ml MCP-1 for 10 min. Protein extraction and other procedures were as described above.

### Cellular imaging

HEK293 cells were cultured on poly-L-lysine-coated cover glass and transfected with plasmids containing epitope-tagged CCR2 isoforms. After 36 h, the cells were fixed with 4% paraformaldehyde solution for 10 min. After washing with PBS, the cells were exposed to 4′,6-diamidino-2-phenylindole (DAPI). The GFP signals were observed under a confocal microscope (LSM800, Carl Zeiss Microimaging Inc., Zena, Germany).

### Reporter gene assay

HEK293 cells expressing exogenous Gαqi were seeded in 24-well plates at a density of 8 × 10^4^ cells/well. The next day, a mixture containing 200 ng pcDNA3.1-CCR2, 200 ng SRE-Luc reporter (firefly luciferase) gene, 50 ng pRL-TK plasmids carrying Renilla luciferase gene, and 1 µl Lipofectamine 2000 was added to each well, according to the manufacturer’s instructions. After approximately 48 h of transfection, overnight-starved cells were treated with 100 ng/ml MCP-1 for 6 h. Cells were then lysed with 100 µl lysis buffer, and the luciferase activity of the cell extract was measured using a luciferase assay system, according to the standard protocol for the Synergy 2 Multi-Mode Microplate Reader (BioTek, USA). The luciferase activities of all groups were normalized with the activities from Renilla luciferase.

### Growth assay

HeLa cells lacking CCR2 were seeded at a density of 4 × 10^3^ cells per well in four different 96-well plates. After 1, 2, 3, or 4 days, 10 µl of cell counting kit 8 (CCK-8, Dojindo Molecular Technologies, Inc., Rockville, MD, USA) solution was added to each well of one plate and incubated at 37 °C for 2 h. The optical density at 450 nm was measured with a microplate reader to determine cell growth. The cells in the other plates were incubated with DMEM containing 1% FBS with or without 100 ng/ml MCP-1 for 24 h. Cell growth was measured using a CCK-8 kit following the manufacturer’s instructions.

### Migration assay

The chemotaxis assay was performed using CCR2 KO HeLa cells, 8-µm pore filters (Corning Inc. Corning, NY, USA), and 24-well Transwell plates. A 650 µl volume of DMEM containing 1% FBS with or without MCP-1 (100 ng/ml) was added to the lower chamber of the Transwell plates. In this experiment, HeLa cells were maintained in the media containing 1% FBS, because they were vulnerable without serum. Cells were loaded into the upper chamber at a density of 1 × 10^4^ cells in 100 µl DMEM and incubated at 37 °C in a 5% CO_2_ incubator. After 24 h, the non-migrated cells retained on the upper side were removed by wiping with a cotton swab. Cells that successfully migrated through the filter were fixed with methanol, stained with hematoxylin and eosin, and counted from four randomly selected optical microscopic fields (100× objective).

### Statistical analysis

Statistical analysis was performed with an unpaired Student’s *t*-tests or analysis of variance using PRISM software (GraphPad; La Jolla, CA, USA). Group means were analyzed using Bonferroni’s multiple comparison tests. Data are presented as the mean ± standard deviation, and all experiments were performed in triplicate unless otherwise indicated.

## Supplementary Information

**Additional file 1.** Supplementary figures.

## Data Availability

Please contact the corresponding author for data, which will be provided on reasonable request.

## Abbreviations

CCR2: C–C chemokine receptor 2
MCP-1: Monocyte chemoattractant protein-1
GRK: G protein-coupled receptor kinase
GPCR: G protein-coupled receptor
NanoBiT: NanoLuc Binary Technology
GFP: Green fluorescent protein
SRE: Serum response element
