# Supplementary material for: Analysis of CCR2 splice variant expression patterns and functional properties
Source: Cell Biosci. 2022 May 12;12:59. doi: 10.1186/s13578-022-00787-6 (PMC9102224; doi:10.1186/s13578-022-00787-6)
Supplement: Supplementary file 1 — Additional file 1. Supplementary figures. [file 13578_2022_787_MOESM1_ESM.pdf]

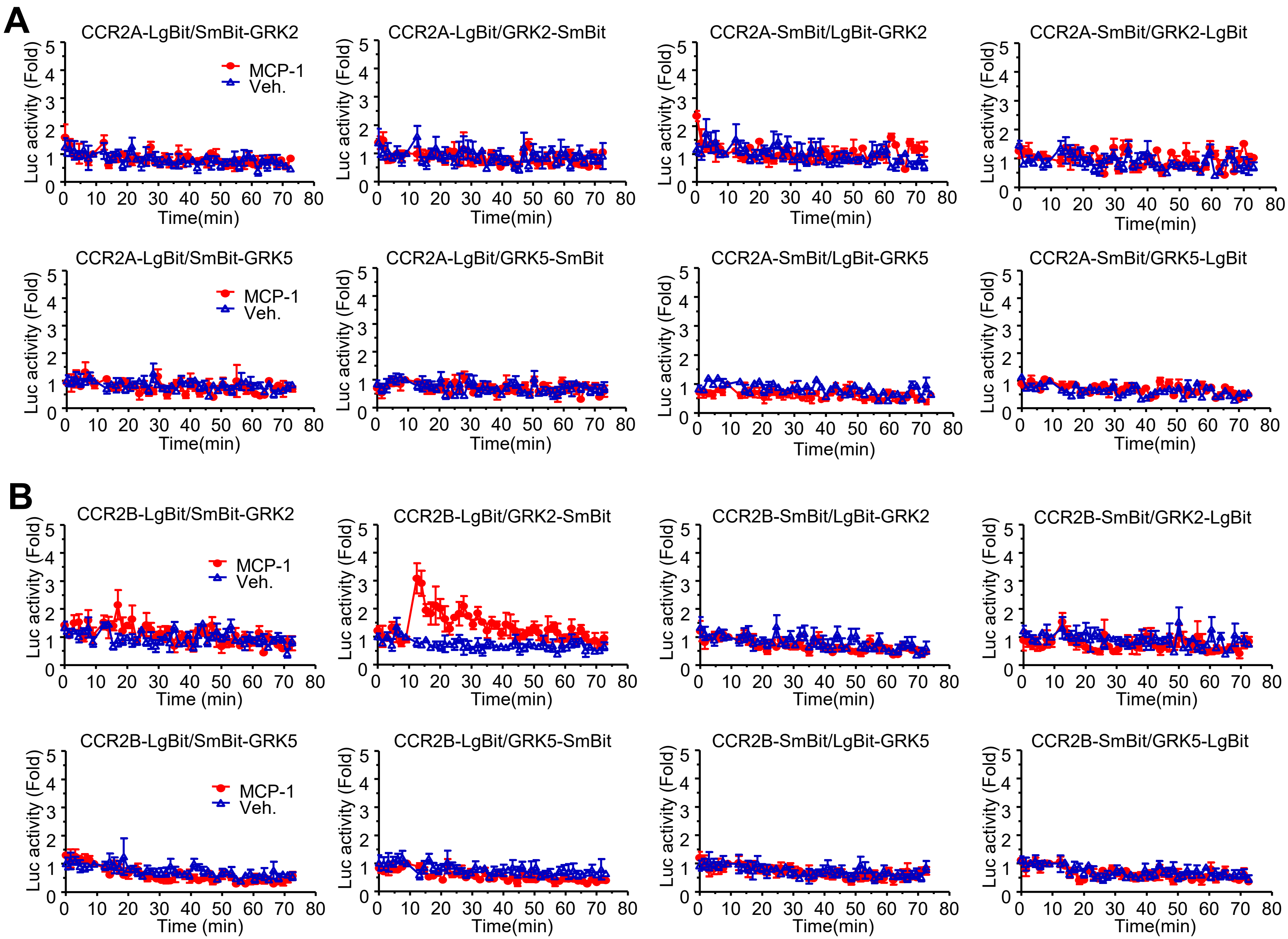

Fig. S1 Ligand-stimulated real-time luciferase activities in HEK293 cells expressing different combination of NanoBit constructs of CCR2 variants and GRKs

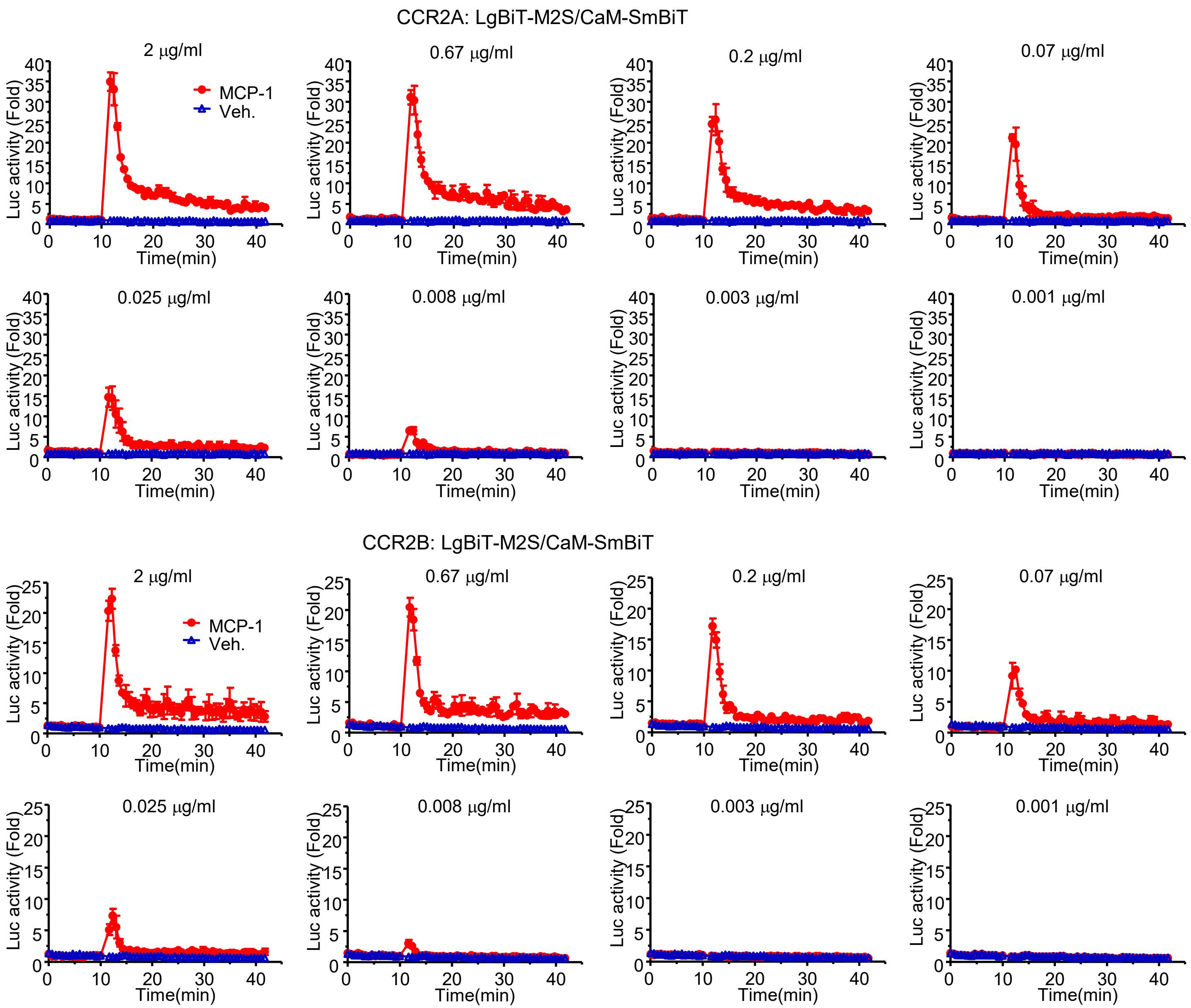

Fig. S2 MCP-1 stimulated intracellular calcium responses figured out by luciferase activity. HEK293-Gqi cells expressing CCR2 variant and calcium probes were treated with difference doses of MCP-1 and luminescence signals were measured by a luminometer.
